# Functional capacity improvement and cardiovascular events after lower extremity revascularization

**DOI:** 10.1371/journal.pone.0357038

**Published:** 2026-09-01

**Authors:** Sean P. Heffron, Yuhe Xia, Crystalann Rodriguez, Caron B. Rockman, Jeffrey S. Berger

**Affiliations:** 1 Leon H. Charney Division of Cardiology, NYU Grossman School of Medicine, New York, New York, United States of America; 2 NYU Center for the Prevention of Cardiovascular Disease, NYU Grossman School of Medicine, New York, New York, United States of America; 3 Division of Biostatistics, NYU Grossman School of Medicine, New York, New York, United States of America; 4 Department of Vascular Surgery, Hackensack University Medical Center, Hackensack, New Jersey, United States of America; 5 Department of Surgery, NYU Grossman School of Medicine, New York, New York, United States of America; NIHR Leicester Biomedical Research Centre, UNITED KINGDOM OF GREAT BRITAIN AND NORTHERN IRELAND

## Abstract

**Background:**

Functional status predicts outcomes in peripheral artery disease (PAD), but the relevance of changes in functional capacity after lower extremity revascularization (LER) and long-term events is unclear.

**Methods:**

In an IRB-approved prospective cohort, we evaluated adjudicated major adverse cardiovascular and limb events (MACLE: death, myocardial infarction, stroke, major amputation, or acute limb ischemia requiring revascularization) among patients with severe PAD undergoing LER. Functional capacity was assessed using the Duke Activity Status Index (DASI) prior to and at 6 months post-LER. Participants were categorized as: Lower-to-Lower (DASI below cohort pre-LER median [15.95] at both time points), Higher-to-Higher (above median at both), or Lower-to-Higher (below at baseline, above at 6 months). Landmark analyses examined MACLE and all-cause mortality from 6 months onward, with Cox models adjusted for demographic and clinical covariates.

**Results:**

Overall, 143 participants had DASI data from before and 6 months after LER (median age 74 years; 36.4% female; 40.6% non-White). Baseline ankle-brachial index (0.56 [0.41, 0.82]) did not differ between groups. The Lower-to-Higher group (n = 28) increased DASI by 17.0 [11.8, 27.0] points (~2.1 METs). MACLE (p < 0.01) and mortality (p < 0.05) differed significantly across groups. Persistently lower DASI was associated with higher adjusted risks of MACLE (HR = 2.03, p = 0.09) and death (HR = 6.78, p = 0.01) relative to persistently higher DASI. Patients whose DASI improved (Lower-to-Higher) had risk similar to those who sustained DASI above the cohort pre-LER median (Higher-to-Higher group).

**Conclusions:**

Among patients with severe PAD undergoing LER with very low DASI, failure to improve functional capacity at 6 months post-LER was associated with higher rates of long-term MACLE and mortality, whereas patients making even modest gains in functional capacity post-LER experienced outcomes comparable to those with consistently greater than cohort average function.

**Trial Registration:** ClinicalTrials.gov NCT02106429.

## Introduction

Functional status is a powerful predictor of clinical outcomes in patients with peripheral artery disease (PAD) [1]. Lower extremity revascularization (LER) is commonly performed in symptomatic PAD to improve blood flow, limb outcomes and quality of life, although patients often remain at increased risk of cardiovascular (CV) events [2]. Limited data suggest that improvements in measures of CV fitness correlate with improved CV outcomes [3], especially among those with low fitness. However, whether improvements in functional capacity following LER associate with better clinical outcomes is uncertain.

## Methodology

Men and women scheduled for LER procedures at NYU Tisch Hospital or Bellevue Hospital Center (New York, NY) provided written informed consent and were enrolled from February 28, 2014 to September 28, 2015 into an IRB-approved protocol (NCT02106429) conforming to the Declaration of Helsinki. Duke Activity Score Index (DASI) questionnaires were completed at baseline (prior to LER) and 6 months following LER. Clinical follow-up of subjects occurred prospectively at 30-days and then every 6 months from the time of LER with adjudication of all major adverse CV and limb events (MACLE – defined as death, myocardial infarction (MI), stroke, major amputation, and acute limb ischemia requiring revascularization). We performed a landmark analysis of MACLE occurring from 6-months post-LER onward. For the purposes of this analysis, we equally divided the cohort at the baseline median DASI [15.95]. We then divided these groups based upon whether their 6-month follow-up DASI was above or below the baseline median.

Comparisons of baseline characteristics of each group were performed with linear regression. Cumulative event rates and Kaplan-Meier curves for mortality and MACLE were calculated based upon DASI group and compared using log-rank tests. Hazard ratios were adjusted for age, sex, smoking, ethnicity, body mass index, prior myocardial infarction, history of heart failure, history of diabetes, and presence of critical limb ischemia. JSB had full access to all the data in the study and takes responsibility for its integrity and the data analysis.

## Results

**Fig 1:**
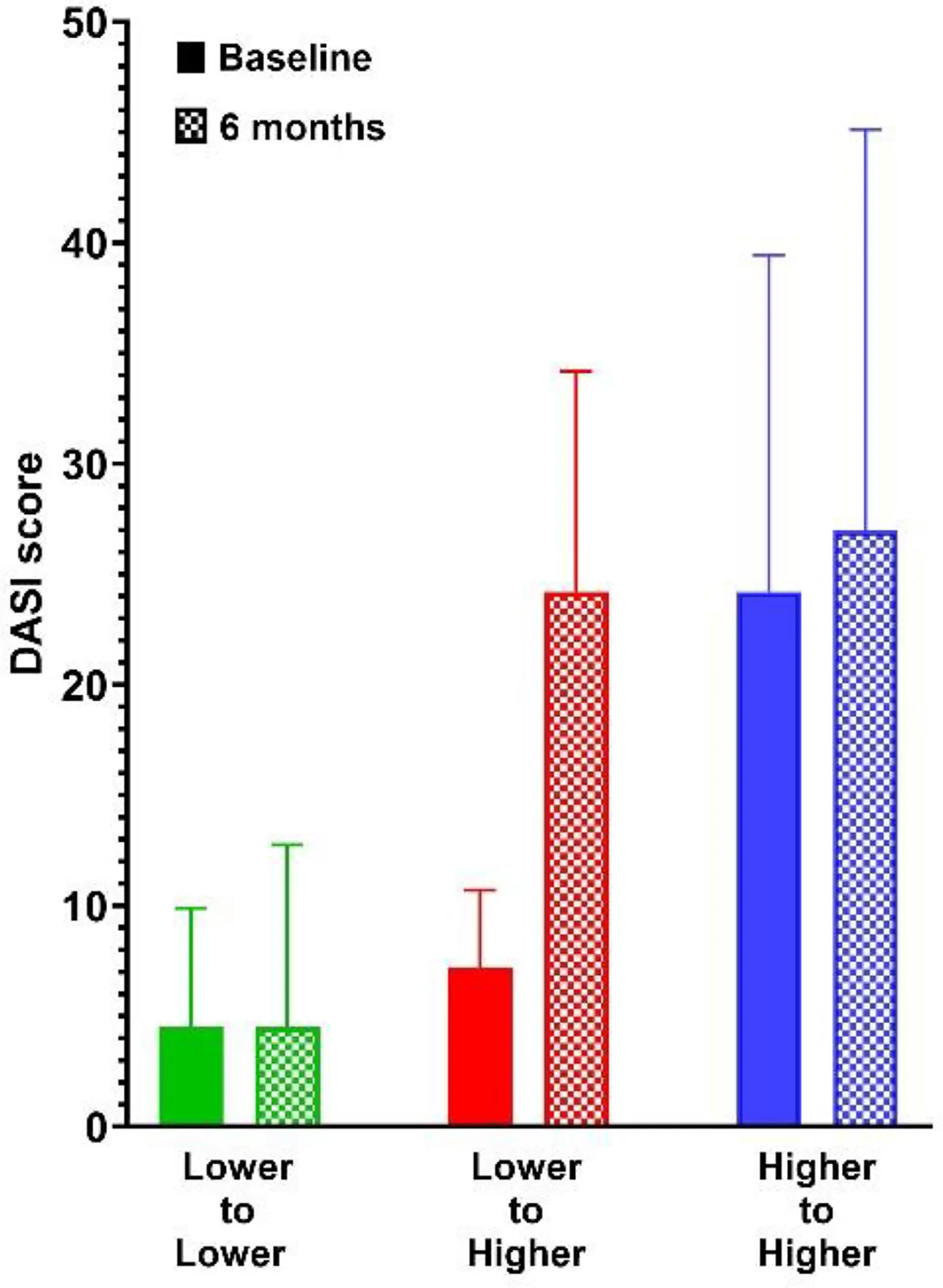
Median [IQR] DASI scores prior to (baseline) and 6 months following LER. Lower-to-Lower DASI – DASI score below the cohort baseline median before and at 6 months post-LER. Higher-to-Higher DASI – DASI score above the cohort baseline median before and at 6 months post-LER. Lower-to-Higher DASI – DASI score increased from below (pre-LER) to above (at 6 months post-LER) the cohort baseline median. DASI – Duke Activity Status Index. IQR – Interquartile range. LER – Lower extremity revascularization.

Overall, 143 participants (74 [67, 82] years, 36.4% female, 40.6% non-White) had DASI scores at baseline and 6-months. Pre-LER ABI of the cohort was 0.57 [0.42, 0.97] and did not differ between groups. Sixty-three patients underwent open revascularization, 66 endovascular, and 14 hybrid revascularization. Extended description of the cohort characteristics can be found in Table 1. The cohort median DASI prior to LER was 15.95. Participants were divided into 3 groups (Table 1, Fig 1): 1) Lower-to-Lower DASI (both pre- and post-LER DASI scores below baseline cohort median; n = 50); 2) Higher-to-Higher DASI (both pre- and post-LER DASI scores above baseline cohort median; n = 65); and 3) Lower-to-Higher DASI (pre-LER DASI score below baseline median that increased to above baseline median at 6-months post-LER; n = 28). The average increase in DASI within the Lower-to-Higher DASI group was 17.0 [11.8, 27.0]

**Table 1:** Characteristics of the cohort stratified by Duke Activity Score Index.

		Overall	Higher-to-Higher	Lower-to-Higher	Lower-to-Lower	p-value
**N =**		143	65	28	50	
**Age, years (median [IQR])**		74 [67, 82]	74 [67, 77]	73 [66, 78]	79 [68, 86]	0.02
**Female sex (%)**		52 (36.4)	17 (26.2)	6 (21.4)	29 (58.0)	<0.001
**Race (%)**	**White**	85 (59.4)	48 (73.8)	17 (60.7)	20 (40.0)	0.01
	**Black**	33 (23.1)	9 (13.8)	7 (25.0)	17 (34.0)	
	**Other**	25 (17.5)	8 (12.3)	4 (14.3)	13 (26.0)	
**Hispanic Ethnicity (%)**		26 (18.3)	8 (12.3)	3 (11.1)	15 (30.0)	0.04
**Body mass index, kg/m**^**2**^ **(median [IQR])**		26.5 [23.3, 29.9]	27.0 [23.2, 29.5]	26.0 [23.6, 30.7]	27.0 [23.6, 31.3]	0.70
**Current tobacco use (%)**		24 (16.8)	15 (23.1)	5 (17.9)	4 (8.0)	0.09
**Family History of ASCVD (%)**		48 (33.6)	30 (46.2)	8 (28.6)	10 (20.0)	<0.01
**Coronary artery disese (%)**		72 (50.3)	30 (46.2)	16 (57.1)	26 (52.0)	0.60
**History of myocardial infarction (%)**		35 (24.5)	9 (13.8)	11 (39.3)	15 (30.0)	0.02
**History of stroke/TIA (%)**		21 (14.7)	8 (12.3)	4 (14.3)	9 (18.0)	0.64
**Carotid artery diseae (%)**		19 (13.3)	5 (7.7)	7 (25.0)	7 (14.0)	0.08
**Diabetes (%)**		70 (49.0)	21 (32.3)	14 (50.0)	35 (70.0)	<0.001
**Hypertension (%)**		128 (89.5)	57 (87.7)	27 (96.4)	44 (88.0)	0.49
**Hyperlipidemia (%)**		107 (74.8)	51 (78.5)	22 (78.6)	34 (68.0)	0.39
**Heart failure (%)**		26 (18.2)	6 (9.2)	6 (21.4)	14 (28.0)	0.03
**Procedure type (%)**	**Endovascular**	66 (46.2)	32 (49.2)	8 (28.6)	26 (52.0)	0.31
	**Open**	63 (44.1)	27 (41.5)	17 (60.7)	19 (38.0)	
	**Hybrid**	14 (9.8)	6 (9.2)	3 (10.7)	5 (10.0)	
**Intermittent claudication (%)**		87 (45.5)	27 (41.5)	14 (50.0)	24 (48.0)	0.68
**CLI indication for LER (%)**		113 (79.0)	44 (67.6)	21 (75.0)	48 (96.0)	0.04
**Aspirin use (%)**		112 (78.3)	56 (86.2)	23 (82.1)	33 (66.0)	0.03
**ABI (median [IQR])**		0.56 [0.41, 0.82]	0.60 [0.42, 0.78]	0.53 [0.42, 0.82]	0.54 [0.42, 1.00]	0.93
**Any antiplatelet use (%)**		125 (87.4)	60 (92.3)	27 (96.4)	38 (76.0)	0.01
**Clopidogrel use (%)**		56 (39.2)	23 (35.4)	13 (46.4)	20 (40.0)	0.60
**Statin use (%)**		115 (80.4)	54 (83.1)	24 (85.7)	37 (74.0)	0.36

**Legend:** ABI – ankle-brachial index; ASCVD – atherosclerotic cardiovascular disease; CLI – critical limb ischemia; IQR – interquartile range; TIA – transient ischemic attack.

**Fig 2:**
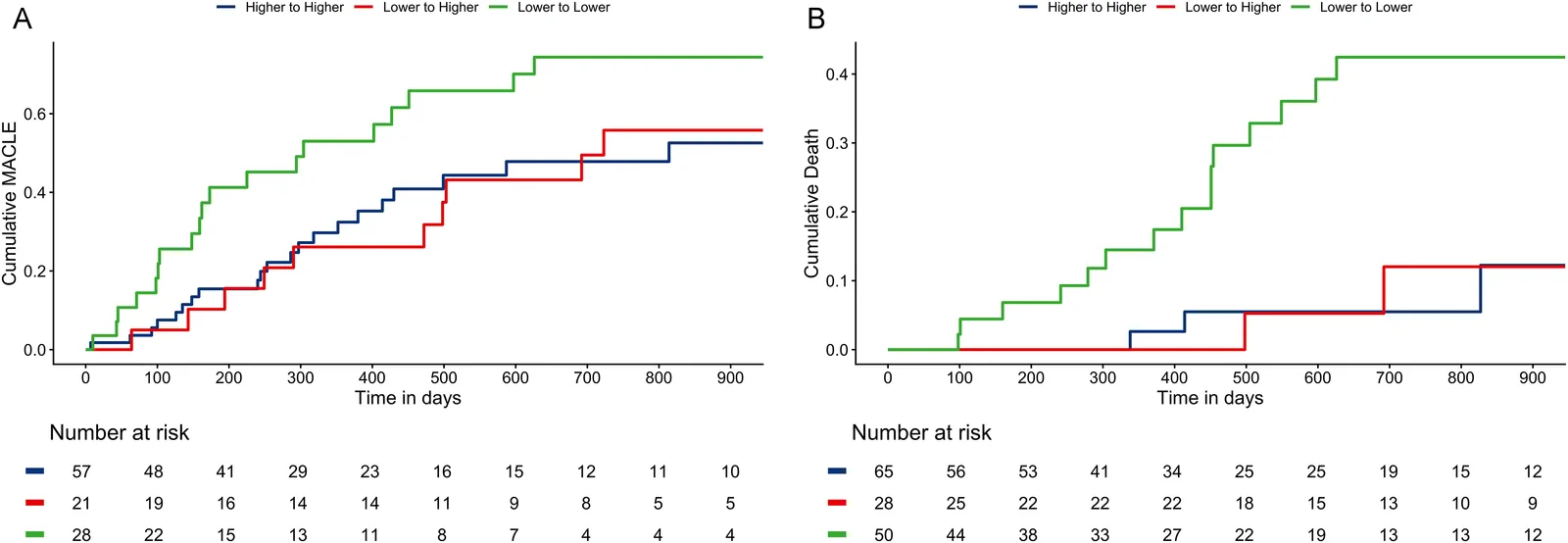
Unadjusted landmark Kaplan-Meier event curves for MACLE (a) and all-cause death (b) from 6-months post-LER. Lower-to-Lower DASI – DASI score below the cohort baseline median before and at 6 months post-LER. Higher-to-Higher DASI – DASI score above the cohort baseline median before and at 6 months post-LER. Lower-to-Higher DASI – DASI score increased from below (pre-LER) to above (at 6 months post-LER) the cohort baseline median. DASI – Duke Activity Status Index; LER – Lower extremity revascularization; MACLE – Major adverse cardiovascular and limb events.

**Fig 3:**
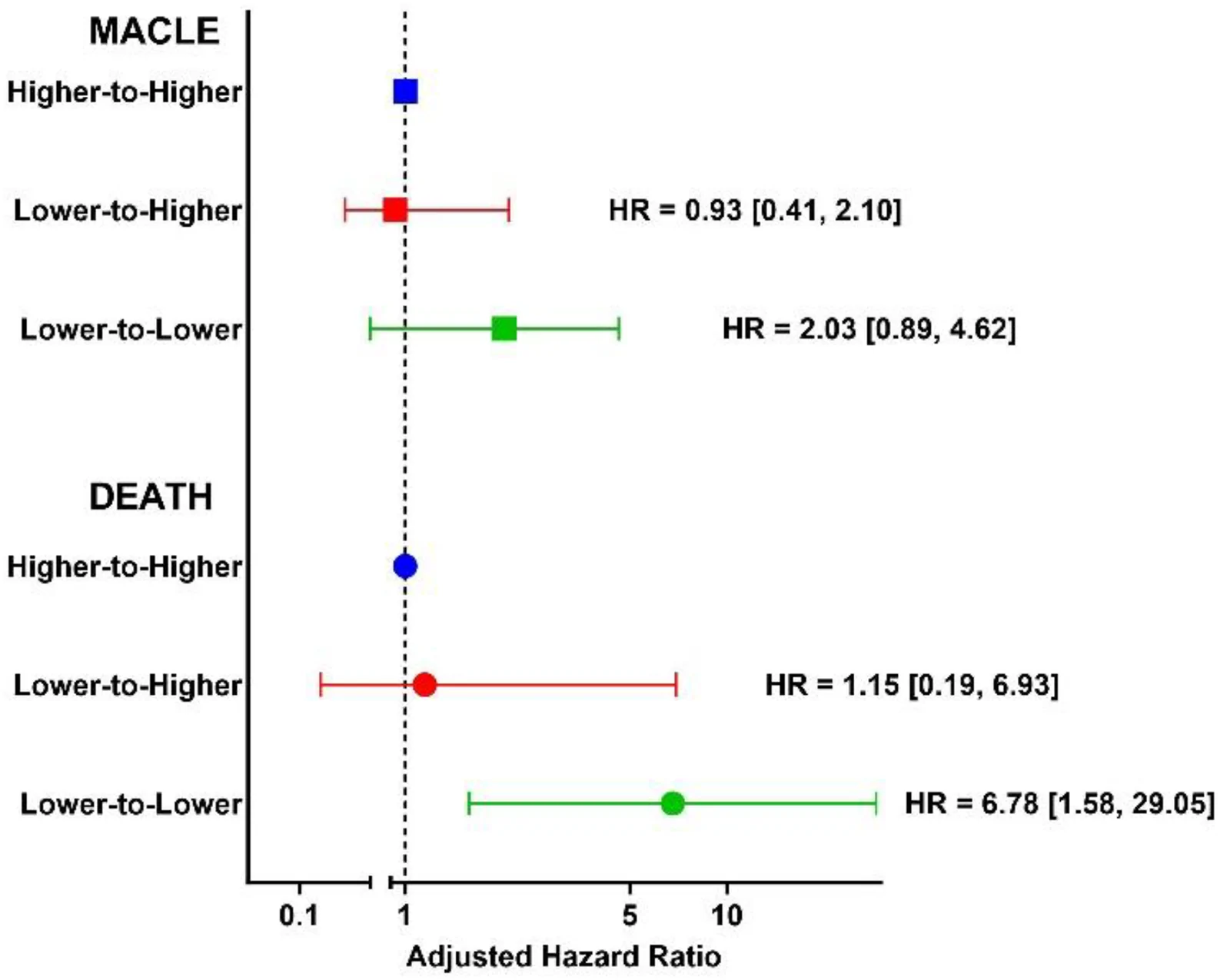
Adjusted hazard ratios for MACLE and death relative to patients with High-to-High DASI scores. Adjusted for age, sex, race, smoking, ethnicity, body mass index, prior MI, heart failure, diabetes and critical limb ischemia. Lower-to-Lower DASI – DASI score below the cohort baseline median before and at 6 months post-LER. Higher-to-Higher DASI – DASI score above the cohort baseline median before and at 6 months post-LER. Lower-to-Higher DASI – DASI score increased from below (pre-LER) to above (at 6 months post-LER) the cohort baseline median. DASI – Duke Activity Status Index; HR – Hazard Ratio; MACLE – Major adverse cardiovascular and limb events; MI – Myocardial infarction.

Figure 2 displays unadjusted Kaplan-Meier curves across the 3 groups. There were significant trends for different rates of MACLE (p < 0.01) and death (p < 0.05) across groups. After adjustment for age, sex, race, ethnicity, smoking, body mass index, prior MI, heart failure, diabetes and critical limb ischemia, participants with DASI persistently lower than cohort baseline median had higher risk of MACLE and death than those with DASI above the baseline median before and 6-months after LER (p = 0.09 and p = 0.01, respectively). Importantly, participants with an increase in DASI from below to above the baseline median at 6-months post-LER exhibited similar risk of MACLE and all-cause mortality as those with DASI consistently above the cohort baseline median (Figs 2 and 3).

## Discussion

Our findings suggest that functional capacity prior to and at 6-months following LER are strongly associated with clinical outcomes in patients with severe PAD over several years following LER. Patients with lower than average for the cohort functional capacity pre-procedure who failed to improve post-procedure were at significantly higher risk of adverse outcomes than all other patients. However, the approximately 1 in 5 subjects with lower than median pre-LER functional capacity who exhibited an increase in DASI to above the population baseline median at 6-months post-LER had outcomes no different from those individuals with higher functional capacity pre-LER. Identifying factors that determine who experiences an increase in functional capacity following LER is an important goal, given that this is a modifiable feature that is associated with cardiac and limb events and especially all-cause mortality. Potential factors may include the extent of vascular disease, comorbid conditions, and adherence to post-procedure rehabilitation programs.

As expected, our cohort of patients with severe PAD undergoing LER had very low baseline functional capacity (median DASI 15.95, equivalent to 4.7 METs), reflecting the high burden of advanced PAD in the study population – in accordance with the high prevalence of critical limb ischemia. Notably, the median DASI of our population is similar to DASI values in Quartile 1 (<18.95) of a prior report of stable PAD and CV outcomes [4]. As in our study, the lowest DASI quartile in this earlier report experienced a greater incidence of adverse outcomes (2.5 times) than those with the highest DASI scores in Quartile 4. To our knowledge, no prior studies have assessed outcomes in PAD post-LER stratified by post-procedural changes in functional capacity. Our study reiterates the very high risk of adverse CV outcomes among patients with PAD and very low DASI, but moves beyond this one-time measure of DASI by demonstrating the potential improvement in prognosis associated with increasing DASI from very low levels among patients with severe PAD. A related observation can be found in a study of participants in cardiac rehabilitation [5] in which low pre-program heart rate recovery (HRR) – indicative of poor CV fitness – was associated with higher mortality, while patients who normalized HRR post-rehabilitation experienced mortality rates similar to those with normal pre-rehabilitation HRR. This underscores the importance of targeted interventions to enhance functional capacity in individuals undergoing procedures to address atherosclerotic CV disease. The potential impact of small changes in functional capacity should serve to motivate efforts in this regard. At baseline, the recorded DASI within our cohort were very low (median ~16). Nonetheless, even small increases following LER among individuals with lower-than-average functional capacity prior to revascularization were associated with significantly lower events – consistent with the potential for improved outcomes with even modest increases in functional capacity [3].

Improvements in CV fitness are elicited by increases in regular physical activity. Whether it is fitness itself, or anti-atherothrombotic processes stimulated by greater physical activity [6] that are responsible for better outcomes, is uncertain. Further, the mechanisms responsible for the improved CV outcomes associated with exercise are incompletely characterized. Prospective study of changes in functional capacity in individuals with PAD, may lend valuable insight into novel interventions to improve outcomes in this high-risk population.

Clinically, our findings suggest that functional recovery after LER may provide prognostic information beyond procedural success alone. Serial functional assessment may help identify patients with persistently low post-procedural capacity who could benefit from closer follow-up, optimization of guideline-directed medical therapy, reassessment for residual or recurrent limb ischemia, and referral for rehabilitation or mobility-focused interventions. However, these findings should be considered hypothesis-generating and require validation in larger contemporary cohorts before being incorporated into routine decision-making algorithms.

Several limitations to our study merit consideration. First, this was an exploratory single-region cohort study with enrollment from 2014 to 2015, which may affect contemporary generalizability. Second, participants were required to have both baseline and 6-month DASI assessments, and incomplete follow-up may have introduced selection bias. In particular, patients with worse health status or more severe PAD may have been less likely to complete repeat functional assessments or die less than 6-months after LER, potentially influencing both group assignment and observed associations. Third, the cohort median DASI was used as an internal threshold to stratify this PAD cohort according to relative functional capacity and to evaluate trajectories of functional change over time using balanced, data-driven groups, and thus should not be interpreted as a validated clinical cutoff for operative fitness. Of note, a DASI of 10 is felt to be approximately equivalent to 4 METS, a common measure of peri-procedural risk [7,8]. Fourth, PAD severity may confound both baseline functional status and subsequent outcomes, and although our adjusted analyses included critical limb ischemia, residual confounding by disease severity remains possible. Finally, the high prevalence of chronic limb-threatening ischemia in this cohort may limit generalizability to patients with less severe PAD.

## Conclusion

Among patients with severe PAD undergoing LER, improvement in very low functional capacity over the first 6 months after the procedure was associated with long-term risks of MACLE and all-cause mortality equivalent to those individuals with greater than the median functional capacity pre-LER, whereas persistently lower functional status identified a particularly high-risk group. These findings suggest serial functional assessment after LER could be a potentially valuable prognostic tool and provide rationale for larger contemporary studies to confirm whether targeted strategies that improve post-procedural functional recovery can translate into better clinical outcomes.
