# Supplementary material for: Functional capacity improvement and cardiovascular events after lower extremity revascularization
Source: PLoS One. 2026 Sep 1;21(9):e0357038. doi: 10.1371/journal.pone.0357038 (PMC13533372; doi:10.1371/journal.pone.0357038)
Supplement: Supplementary file 1 [file pone.0357038.s001.docx]

Reviewer #1:

1) The cutoff between 'fit' and 'non-fit' is arbitrary (based on the median DASI of the cohort), with no reference to traditional surgical fitness cutoffs (eg, DASI of 34, 4 METs);

We thank the Reviewer for this important comment and agree that the use of the cohort median as the threshold for categorization requires justification. Our intention was not to define clinical “fitness” or “non-fitness” according to an established perioperative standard, but rather to stratify this PAD cohort according to relative functional capacity and to evaluate trajectories of functional change over time using balanced, data-driven groups.

We agree that this median-based approach is cohort-specific and should not be interpreted as equivalent to validated surgical fitness thresholds such as a DASI corresponding to approximately 4 METs. In response, we have revised the manuscript to clarify that the cutoff was used as an analytic stratification method rather than a clinically established definition of operative fitness. We have adjusted our terminology from “low” and “high” to “lower” and “higher.” We have also added discussion of conventional DASI/MET-based thresholds in the Discussion.

Importantly (and not surprising), our cohort of PAD patients undergoing lower extremity revascularization had very low baseline functional capacity overall (median DASI 15.95), reflecting the high burden of advanced PAD in the study population. The median DASI amongst our population is similar to DASI values in Quartile 1 (<18.95) in Senthong et al.’s report of stable PAD and CV outcomes (PMID: 27892883). As in our study, the patients in this lowest DASI quartile experienced ~250% greater adverse outcomes than those in Quartile 4. Our study moves beyond this one-time measure of DASI and adds to the literature by demonstrating the potential improvement in prognosis associated with increasing DASI (even a small amount) from very low levels among patients with severe PAD. We now include mention of the above within our revised Discussion.

We believe this supports the value of internal cohort stratification for this exploratory analysis, while still recognizing that external clinical thresholds may be more relevant for perioperative decision-making in other settings.

**Revisions made:**

- Clarified in the Methods that the baseline median DASI was used for internal cohort stratification, not as a validated clinical cutoff.
- Added discussion acknowledging conventional DASI thresholds and the limitation of using a cohort-derived median.
- Added discussion of the relevance of our cohort median DASI within the context of the study by Senthong et al.

2) There is no 'high-to-low' fitness group. It would be anticipated that some patients would decline post revascularisation;

We agree with the reviewer that decline in functional status after revascularization is clinically plausible and important, particularly in a population with substantial comorbidity and advanced PAD. To address this concern, we include below our analyses including this small subgroup (light blue line). As you can see, outcomes in this group were numerically better than the highest risk group (Lower-to-Lower; dark blue) and numerically worse than the lowest risk group (Higher-to-Higher; green).

We now acknowledge in the Discussion that post-LER functional decline may occur and could reflect worsening comorbidity, recurrent limb symptoms, incomplete recovery, frailty progression, or interval cardiovascular events. We highlight in the discussion that this subgroup warrants dedicated study in larger cohorts.

**Revisions made:**

- Added discussion acknowledging the clinical relevance of post-LER decline in functional capacity and the need for larger studies to assess this trajectory.

3) In relation to the comment above, could the revascularisation procedures be briefly specified (eg, were they all treated by endovascular means);

Among the revascularization procedures, 48.3% were endovascular, 44.8% were open and the remaining 6.9% were hybrid. This breakdown is included in the Table within the revised manuscript.

4) what variables were included in the multivariable cox regression.

Age, sex, race, ethnicity, smoking, body mass index, prior MI, heart failure, diabetes and critical limb ischemia were included in the fully-adjusted Cox regression. These variables are listed in the caption for Figure 3 and also in the revised Methodology section

Reviewer #2: The authors present data from a prospective study undertaken in 2014 - 2015 recruiting 151 patients undergoing lower limb revascularisation for chronic peripheral artery disease. Patients underwent functional status assessment using the Duke Activity Score Index at baseline and at 6 months post-revascularisation. DASI scores were dichotomised at the median at baseline and patients were categorised into three groups - those that had DASI scores
My main concern regarding this manuscript is its brevity. It appears to be written as a research letter rather than a full paper. The data is also quite old and follow-up relatively incomplete (numbers at risk are very low even at 1 year suggesting significant loss to follow-up).

We thank the Reviewer for this helpful assessment. We agree that the original version which was submitted as a letter was very concise and did not provide sufficient detail regarding cohort composition, analytic choices, follow-up, and interpretation. In response, we have expanded the manuscript substantially. Specifically, we added clarifying detail regarding the rationale for DASI categorization, clinical characteristics of the cohort, the high prevalence of chronic limb-threatening ischemia (CLTI), and the potential clinical implications of the findings.

We agree that both the age of the dataset and amount of incomplete follow-up bear mention. We have revised the manuscript to explicitly note that enrollment occurred from February 2014 to September 2015. While the dataset is not necessarily contemporary, we believe the question addressed remains clinically relevant, namely whether change in functional capacity after lower extremity revascularization is associated with subsequent cardiovascular and limb outcomes.

We also point out that in contrast to suffering from what may have appears as a high rate of loss to follow-up, the cohort actually experienced very high composite of cardiac and limb events (58% MACLE incidence over median follow-up of 19 months post-LER).

In the revised Discussion, we also acknowledge that requiring both baseline and 6-month DASI data may have introduced selection bias as patients experiencing MACLE prior to 6-months post-LER were not included.

**Revisions made:**

- Acknowledge the 2014–2015 study period in the Methodology section.
- Explain numbers of patients at follow-up is secondary to high event rates, not attrition/LTFU

The findings are interesting, although not unexpected, in that those with higher functional status at 6 months have better outcomes. I suspect a major confounder here is severity of PAD - there is not any mention of proportion of patients in the sample with intermittent claudication vs those with CLTI.

We thank the Reviewer for highlighting this important issue. We agree that severity of PAD is a key potential confounder. The original manuscript noted adjustment for critical limb ischemia in the multivariable analysis, but did not adequately report the clinical severity distribution of the cohort.

In response, we have revised the manuscript to report that the overall proportion of patients with CLTI was high. We also now provide the proportion with intermittent claudication versus CLTI in the cohort, and where available, by revascularization type. This contextualizes the low baseline DASI values and emphasizes that the cohort largely comprised patients with advanced limb ischemia rather than a broad-spectrum PAD population.

We also now discuss more explicitly that PAD severity may confound both baseline functional status and subsequent outcomes, and that although our adjusted analyses included critical limb ischemia, residual confounding by disease severity remains possible.

To address the reviewer’s concern about confounding by the presence or absence of CLI as the LER indication within the different groups, we repeated analyses including only patients with CLI referred for LER. This did not alter the overall findings as shown in the recreated Figures 2 and 3 (only including patients presenting with CLI) below.

**Revisions made:**

- Added the proportions of intermittent claudication and CLTI to the Results.
- Expanded the Discussion to address PAD severity as a major potential confounder.
- Clarified that adjustment for CLTI does not eliminate the possibility of residual confounding.

I would prefer this paper written up as a full research article. This would give the authors more space to discuss their methods and results more fully. Additionally it requires a more in-depth discussion to explain the results. The authors should discuss loss to follow up, proportions of patients with intermittent claudication vs CLTI. Additionally the authors should discuss the clinical relevance of their findings - how should clinicians use these data to inform decision making with their patients?

We appreciate this thoughtful suggestion and agree that the clinical interpretation needed to be better developed. In the revised manuscript, we have expanded the Discussion substantially to address the issues raised as described above. We now emphasize that our findings should be viewed as hypothesis-generating and not as evidence that DASI alone should guide revascularization decisions. Rather, serial functional assessment may provide a useful patient-centered adjunct to routine follow-up after LER. Specifically, the data suggest that patients with persistently low functional capacity after revascularization may represent a particularly high-risk subgroup who may benefit from closer surveillance, more intensive secondary prevention, reassessment of limb status, and attention to rehabilitation, mobility, and comorbidity optimization.

We have also revised the manuscript to make clear that procedural success and improved perfusion do not necessarily translate into improved functional recovery, especially in a cohort with a high prevalence of CLTI. Accordingly, measuring functional status longitudinally may help inform prognosis and help set expectations during patient counseling, while future larger studies are needed to determine how best to incorporate such measures into post-LER care pathways.

**Revisions made:**

- Expanded discussion of clinical implications.
- Added explicit discussion of disease severity.
- Clarified that DASI trajectory may serve as a prognostic, patient-centered measure rather than a stand-alone treatment decision tool.
