# Supplementary material for: Functional capacity improvement and cardiovascular events after lower extremity revascularization
Source: PLoS One. 2026 Sep 1;21(9):e0357038. doi: 10.1371/journal.pone.0357038 (PMC13533372; doi:10.1371/journal.pone.0357038)
Supplement: Peer review history

# Peer review: Decision Letter 0

**DOI:** 10.1371/journal.pone.0357038.r001

**Authors:** Athanasios Saratzis

13 Apr 2026

Dear Dr. Heffron,

Please submit your revised manuscript by 31/05/2026. If you will need more time than this to complete your revisions, please reply to this message or contact the journal office at plosone@plos.org. When you're ready to submit your revision, log on to https://www.editorialmanager.com/pone/ and select the 'Submissions Needing Revision' folder to locate your manuscript file.

- A letter that responds to each point raised by the academic editor and reviewer(s). You should upload this letter as a separate file labeled 'Response to Reviewers'.
- A marked-up copy of your manuscript that highlights changes made to the original version. You should upload this as a separate file labeled 'Revised Manuscript with Track Changes'.
- An unmarked version of your revised paper without tracked changes. You should upload this as a separate file labeled 'Manuscript'.

If you would like to make changes to your financial disclosure, please include your updated statement in your cover letter. Guidelines for resubmitting your figure files are available below the reviewer comments at the end of this letter.

If applicable, we recommend that you deposit your laboratory protocols in protocols.io to enhance the reproducibility of your results. Protocols.io assigns your protocol its own identifier (DOI) so that it can be cited independently in the future. For instructions see: https://journals.plos.org/plosone/s/submission-guidelines#loc-laboratory-protocols. Additionally, PLOS ONE offers an option for publishing peer-reviewed Lab Protocol articles, which describe protocols hosted on protocols.io. Read more information on sharing protocols at https://plos.org/protocols?utm_medium=editorial-email&utm_source=authorletters&utm_campaign=protocols.

As the corresponding author, your ORCID iD is verified in the submission system and will appear in the published article. PLOS supports the use of ORCID, and we encourage all coauthors to register for an ORCID iD and use it as well. Please encourage your coauthors to verify their ORCID iD within the submission system before final acceptance, as unverified ORCID iDs will not appear in the published article. *Only*  the individual author can complete the verification step; PLOS staff *cannot*  verify ORCID iDs on behalf of authors.

We look forward to receiving your revised manuscript.

Kind regards,

Athanasios Saratzis

Academic Editor

PLOS One

Journal Requirements:

When submitting your revision, we need you to address these additional requirements.

1. Please ensure that your manuscript meets PLOS ONE's style requirements, including those for file naming. The PLOS ONE style templates can be found at

https://journals.plos.org/plosone/s/file?id=wjVg/PLOSOne_formatting_sample_main_body.pdf and

https://journals.plos.org/plosone/s/file?id=ba62/PLOSOne_formatting_sample_title_authors_affiliations.pdf.

2.  We note that you have indicated that there are restrictions to data sharing for this study. For studies involving human research participant data or other sensitive data, we encourage authors to share de-identified or anonymized data. However, when data cannot be publicly shared for ethical reasons, we allow authors to make their data sets available upon request. For information on unacceptable data access restrictions, please see http://journals.plos.org/plosone/s/data-availability#loc-unacceptable-data-access-restrictions.

Before we proceed with your manuscript, please address the following prompts:

a) If there are ethical or legal restrictions on sharing a de-identified data set, please explain them in detail (e.g., data contain potentially identifying or sensitive patient information, data are owned by a third-party organization, etc.) and who has imposed them (e.g., a Research Ethics Committee or Institutional Review Board, etc.). Please also provide contact information for a data access committee, ethics committee, or other institutional body to which data requests may be sent.

b) If there are no restrictions, please upload the minimal anonymized data set necessary to replicate your study findings to a stable, public repository and provide us with the relevant URLs, DOIs, or accession numbers. Please see http://www.bmj.com/content/340/bmj.c181.long for guidelines on how to de-identify and prepare clinical data for publication. For a list of recommended repositories, please see https://journals.plos.org/plosone/s/recommended-repositories. You also have the option of uploading the data as Supporting Information files, but we would recommend depositing data directly to a data repository if possible.

Please update your Data Availability statement in the submission form accordingly.

3. In the online submission form you indicate that your data is not available for proprietary reasons and have provided a contact point for accessing this data. Please note that your current contact point is a co-author on this manuscript. According to our Data Policy, the contact point must not be an author on the manuscript and must be an institutional contact, ideally not an individual. Please revise your data statement to a non-author institutional point of contact, such as a data access or ethics committee, and send this to us via return email. Please also include contact information for the third party organization, and please include the full citation of where the data can be found.

4. We note that the grant information you provided in the ‘Funding Information’ and ‘Financial Disclosure’ sections do not match.

When you resubmit, please ensure that you provide the correct grant numbers for the awards you received for your study in the ‘Funding Information’ section.

5. Please ensure that you refer to Figure 1 in your text as, if accepted, production will need this reference to link the reader to the figure.

6. If the reviewer comments include a recommendation to cite specific previously published works, please review and evaluate these publications to determine whether they are relevant and should be cited. There is no requirement to cite these works unless the editor has indicated otherwise.

Additional Editor Comments (if provided):

Thank you for your submission the invited reviewers have provided important feedback and comments to be addressed. Please can you address these points in a revision?

[Note: HTML markup is below. Please do not edit.]

Reviewers' comments:

Reviewer's Responses to Questions

**Comments to the Author**

1. Is the manuscript technically sound, and do the data support the conclusions?

Reviewer #1: Yes

Reviewer #2: Yes

**********

2. Has the statistical analysis been performed appropriately and rigorously?

Reviewer #1: Yes

Reviewer #2: Yes

**********

3. Have the authors made all data underlying the findings in their manuscript fully available?

The PLOS Data policy

Reviewer #1: No

Reviewer #2: No

**********

4. Is the manuscript presented in an intelligible fashion and written in standard English?

Reviewer #1: Yes

Reviewer #2: Yes

**********

Reviewer #1: Thank you for submitting this very interesting study that underlines the impact of functional capacity on cardiovascular outcomes. It is well written and has the potential to influence practice, by introducing functional capacity assessments in post-revascularisation pathways. Data are by definition not fully available, due to confidentiality reasons, but this has been reasonably explained. Nonetheless, a few points may be raised: 1) the cutoff between 'fit' and 'non-fit' is arbitrary (based on the median DASI of the cohort), with no reference to traditional surgical fitness cutoffs (eg, DASI of 34, 4 METs); 2) there is no 'high-to-low' fitness group. It would be anticipated that some patients would decline post revascularisation; 3) in relation to the comment above, could the revascularisation procedures be briefly specified (eg, were they all treated by endovascular means); 4) what variables were included in the multivariable cox regression.

Reviewer #2: The authors present data from a prospective study undertaken in 2014 - 2015 recruiting 151 patients undergoing lower limb revascularisation for chronic peripheral artery disease. Patients underwent functional status assessment using the Duke Activity Score Index at baseline and at 6 months post-revascularisation. DASI scores were dichotomised at the median at baseline and patients were categorised into three groups - those that had DASI scores <median 6=“” and=“” at=“” baseline=“” months=“”>

My main concern regarding this manuscript is its brevity. It appears to be written as a research letter rather than a full paper. The data is also quite old and follow-up relatively incomplete (numbers at risk are very low even at 1 year suggesting significant loss to follow-up).

The findings are interesting, although not unexpected, in that those with higher functional status at 6 months have better outcomes. I suspect a major confounder here is severity of PAD - there is not any mention of proportion of patients in the sample with intermittent claudication vs those with CLTI.

I would prefer this paper written up as a full research article. This would give the authors more space to discuss their methods and results more fully. Additionally it requires a more in-depth discussion to explain the results. The authors should discuss loss to follow up, proportions of patients with intermittent claudication vs CLTI. Additionally the authors should discuss the clinical relevance of their findings - how should clinicians use these data to inform decision making with their patients?</median>

**********

what does this mean?). If published, this will include your full peer review and any attached files.

If you choose “no”, your identity will remain anonymous but your review may still be made public.

**Do you want your identity to be public for this peer review?**  For information about this choice, including consent withdrawal, please see our Privacy Policy

Reviewer #1: No

Reviewer #2: **Yes:**  John Houghton

**********

[NOTE: If reviewer comments were submitted as an attachment file, they will be attached to this email and accessible via the submission site. Please log into your account, locate the manuscript record, and check for the action link "View Attachments". If this link does not appear, there are no attachment files.]

To ensure your figures meet our technical requirements, please review our figure guidelines: https://journals.plos.org/plosone/s/figures

You may also use PLOS’s free figure tool, NAAS, to help you prepare publication quality figures: https://journals.plos.org/plosone/s/figures#loc-tools-for-figure-preparation.

NAAS will assess whether your figures meet our technical requirements by comparing each figure against our figure specifications.

# Author comment: Author response to Decision Letter 1

**DOI:** 10.1371/journal.pone.0357038.r002

6 Jul 2026

Reviewer #1:

1) The cutoff between 'fit' and 'non-fit' is arbitrary (based on the median DASI of the cohort), with no reference to traditional surgical fitness cutoffs (eg, DASI of 34, 4 METs);

We thank the Reviewer for this important comment and agree that the use of the cohort median as the threshold for categorization requires justification. Our intention was not to define clinical “fitness” or “non-fitness” according to an established perioperative standard, but rather to stratify this PAD cohort according to relative functional capacity and to evaluate trajectories of functional change over time using balanced, data-driven groups.

We agree that this median-based approach is cohort-specific and should not be interpreted as equivalent to validated surgical fitness thresholds such as a DASI corresponding to approximately 4 METs. In response, we have revised the manuscript to clarify that the cutoff was used as an analytic stratification method rather than a clinically established definition of operative fitness. We have adjusted our terminology from “low” and “high” to “lower” and “higher.” We have also added discussion of conventional DASI/MET-based thresholds in the Discussion.

Importantly (and not surprising), our cohort of PAD patients undergoing lower extremity revascularization had very low baseline functional capacity overall (median DASI 15.95), reflecting the high burden of advanced PAD in the study population. The median DASI amongst our population is similar to DASI values in Quartile 1 (<18.95) in Senthong et al.’s report of stable PAD and CV outcomes (PMID: 27892883). As in our study, the patients in this lowest DASI quartile experienced ~250% greater adverse outcomes than those in Quartile 4. Our study moves beyond this one-time measure of DASI and adds to the literature by demonstrating the potential improvement in prognosis associated with increasing DASI (even a small amount) from very low levels among patients with severe PAD. We now include mention of the above within our revised Discussion.

We believe this supports the value of internal cohort stratification for this exploratory analysis, while still recognizing that external clinical thresholds may be more relevant for perioperative decision-making in other settings.

Revisions made:

• Clarified in the Methods that the baseline median DASI was used for internal cohort stratification, not as a validated clinical cutoff.

• Added discussion acknowledging conventional DASI thresholds and the limitation of using a cohort-derived median.

• Added discussion of the relevance of our cohort median DASI within the context of the study by Senthong et al.

2) There is no 'high-to-low' fitness group. It would be anticipated that some patients would decline post revascularisation;

We agree with the reviewer that decline in functional status after revascularization is clinically plausible and important, particularly in a population with substantial comorbidity and advanced PAD. To address this concern, we include below our analyses including this small subgroup (light blue line). As you can see, outcomes in this group were numerically better than the highest risk group (Lower-to-Lower; dark blue) and numerically worse than the lowest risk group (Higher-to-Higher; green).

We now acknowledge in the Discussion that post-LER functional decline may occur and could reflect worsening comorbidity, recurrent limb symptoms, incomplete recovery, frailty progression, or interval cardiovascular events. We highlight in the discussion that this subgroup warrants dedicated study in larger cohorts.

Revisions made:

• Added discussion acknowledging the clinical relevance of post-LER decline in functional capacity and the need for larger studies to assess this trajectory.

3) In relation to the comment above, could the revascularisation procedures be briefly specified (eg, were they all treated by endovascular means);

Among the revascularization procedures, 48.3% were endovascular, 44.8% were open and the remaining 6.9% were hybrid. This breakdown is included in the Table within the revised manuscript.

4) what variables were included in the multivariable cox regression.

Age, sex, race, ethnicity, smoking, body mass index, prior MI, heart failure, diabetes and critical limb ischemia were included in the fully-adjusted Cox regression. These variables are listed in the caption for Figure 3 and also in the revised Methodology section

Reviewer #2: The authors present data from a prospective study undertaken in 2014 - 2015 recruiting 151 patients undergoing lower limb revascularisation for chronic peripheral artery disease. Patients underwent functional status assessment using the Duke Activity Score Index at baseline and at 6 months post-revascularisation. DASI scores were dichotomised at the median at baseline and patients were categorised into three groups - those that had DASI scores

My main concern regarding this manuscript is its brevity. It appears to be written as a research letter rather than a full paper. The data is also quite old and follow-up relatively incomplete (numbers at risk are very low even at 1 year suggesting significant loss to follow-up).

We thank the Reviewer for this helpful assessment. We agree that the original version which was submitted as a letter was very concise and did not provide sufficient detail regarding cohort composition, analytic choices, follow-up, and interpretation. In response, we have expanded the manuscript substantially. Specifically, we added clarifying detail regarding the rationale for DASI categorization, clinical characteristics of the cohort, the high prevalence of chronic limb-threatening ischemia (CLTI), and the potential clinical implications of the findings.

We agree that both the age of the dataset and amount of incomplete follow-up bear mention. We have revised the manuscript to explicitly note that enrollment occurred from February 2014 to September 2015. While the dataset is not necessarily contemporary, we believe the question addressed remains clinically relevant, namely whether change in functional capacity after lower extremity revascularization is associated with subsequent cardiovascular and limb outcomes.

We also point out that in contrast to suffering from what may have appears as a high rate of loss to follow-up, the cohort actually experienced very high composite of cardiac and limb events (58% MACLE incidence over median follow-up of 19 months post-LER).

In the revised Discussion, we also acknowledge that requiring both baseline and 6-month DASI data may have introduced selection bias as patients experiencing MACLE prior to 6-months post-LER were not included.

Revisions made:

• Acknowledge the 2014–2015 study period in the Methodology section.

• Explain numbers of patients at follow-up is secondary to high event rates, not attrition/LTFU

The findings are interesting, although not unexpected, in that those with higher functional status at 6 months have better outcomes. I suspect a major confounder here is severity of PAD - there is not any mention of proportion of patients in the sample with intermittent claudication vs those with CLTI.

We thank the Reviewer for highlighting this important issue. We agree that severity of PAD is a key potential confounder. The original manuscript noted adjustment for critical limb ischemia in the multivariable analysis, but did not adequately report the clinical severity distribution of the cohort.

In response, we have revised the manuscript to report that the overall proportion of patients with CLTI was high. We also now provide the proportion with intermittent claudication versus CLTI in the cohort, and where available, by revascularization type. This contextualizes the low baseline DASI values and emphasizes that the cohort largely comprised patients with advanced limb ischemia rather than a broad-spectrum PAD population.

We also now discuss more explicitly that PAD severity may confound both baseline functional status and subsequent outcomes, and that although our adjusted analyses included critical limb ischemia, residual confounding by disease severity remains possible.

To address the reviewer’s concern about confounding by the presence or absence of CLI as the LER indication within the different groups, we repeated analyses including only patients with CLI referred for LER. This did not alter the overall findings as shown in the recreated Figures 2 and 3 (only including patients presenting with CLI) below.

Revisions made:

• Added the proportions of intermittent claudication and CLTI to the Results.

• Expanded the Discussion to address PAD severity as a major potential confounder.

• Clarified that adjustment for CLTI does not eliminate the possibility of residual confounding.

I would prefer this paper written up as a full research article. This would give the authors more space to discuss their methods and results more fully. Additionally it requires a more in-depth discussion to explain the results. The authors should discuss loss to follow up, proportions of patients with intermittent claudication vs CLTI. Additionally the authors should discuss the clinical relevance of their findings - how should clinicians use these data to inform decision making with their patients?

We appreciate this thoughtful suggestion and agree that the clinical interpretation needed to be better developed. In the revised manuscript, we have expanded the Discussion substantially to address the issues raised as described above. We now emphasize that our findings should be viewed as hypothesis-generating and not as evidence that DASI alone should guide revascularization decisions. Rather, serial functional assessment may provide a useful patient-centered adjunct to routine follow-up after LER. Specifically, the data suggest that patients with persistently low functional capacity after revascularization may represent a particularly high-risk subgroup who may benefit from closer surveillance, more intensive secondary prevention, reassessment of limb status, and attention to rehabilitation, mobility, and comorbidity optimization.

We have also revised the manuscript to make clear that procedural success and improved perfusion do not necessarily translate into improved functional recovery, especially in a cohort with a high prevalence of CLTI. Accordingly, measuring functional status longitudinally may help inform prognosis and help set expectations during patient counseling, while future larger studies are needed to determine how best to incorporate such measures into post-LER care pathways.

Revisions made:

• Expanded discussion of clinical implications.

• Added explicit discussion of disease severity.

• Clarified that DASI trajectory may serve as a prognostic, patient-centered measure rather than a stand-alone treatment decision tool.

Attachment Submitted filename: PLoS One response to reviewers 06192026.docx

# Editor report: Decision Letter 1

**DOI:** 10.1371/journal.pone.0357038.r003

**Authors:** Athanasios Saratzis

11 Aug 2026

Functional capacity improvement and cardiovascular events after lower extremity revascularization

PONE-D-26-04908R1

Dear Dr. Heffron,

We’re pleased to inform you that your manuscript has been judged scientifically suitable for publication and will be formally accepted for publication once it meets all outstanding technical requirements.

Within one week, you’ll receive an e-mail detailing the required amendments. When these have been addressed, you’ll receive a formal acceptance letter and your manuscript will be scheduled for publication.

An invoice will be generated when your article is formally accepted. Please note, if your institution has a publishing partnership with PLOS and your article meets the relevant criteria, all or part of your publication costs will be covered. Please make sure your user information is up-to-date by logging into Editorial Manager at Editorial Manager® and clicking the ‘Update My Information' link at the top of the page. For questions related to billing, please contact billing support.

If your institution or institutions have a press office, please notify them about your upcoming paper to help maximize its impact. If they’ll be preparing press materials, please inform our press team as soon as possible -- no later than 48 hours after receiving the formal acceptance. Your manuscript will remain under strict press embargo until 2 pm Eastern Time on the date of publication. For more information, please contact onepress@plos.org.

Kind regards,

Athanasios Saratzis

Academic Editor

PLOS One

Additional Editor Comments (optional):

Reviewers' comments:

# Editor report: Acceptance letter

**DOI:** 10.1371/journal.pone.0357038.r004

**Authors:** Athanasios Saratzis

PONE-D-26-04908R1

PLOS One

Dear Dr. Heffron,

I'm pleased to inform you that your manuscript has been deemed suitable for publication in PLOS One. Congratulations! Your manuscript is now being handed over to our production team.

At this stage, our production department will prepare your paper for publication. This includes ensuring the following:

* All references, tables, and figures are properly cited

* All relevant supporting information is included in the manuscript submission,

* There are no issues that prevent the paper from being properly typeset

You will receive further instructions from the production team, including instructions on how to review your proof when it is ready. Please keep in mind that we are working through a large volume of accepted articles, so please give us a few days to review your paper and let you know the next and final steps.

Lastly, if your institution or institutions have a press office, please let them know about your upcoming paper now to help maximize its impact. If they'll be preparing press materials, please inform our press team within the next 48 hours. Your manuscript will remain under strict press embargo until 2 pm Eastern Time on the date of publication. For more information, please contact onepress@plos.org.

You will receive an invoice from PLOS for your publication fee after your manuscript has reached the completed accept phase. If you receive an email requesting payment before acceptance or for any other service, this may be a phishing scheme. Learn how to identify phishing emails and protect your accounts at https://explore.plos.org/phishing.

If we can help with anything else, please email us at customercare@plos.org.

Thank you for submitting your work to PLOS One and supporting open access.

Kind regards,

PLOS One Editorial Office Staff

on behalf of

Dr. Athanasios Saratzis

Academic Editor

PLOS One
